# Acquisition of Dual Ribozyme-Functions in Nonfunctional Short Hairpin RNAs through Kissing-Loop Interactions

**DOI:** 10.3390/life12101561

**Published:** 2022-10-08

**Authors:** Hiromi Mutsuro-Aoki, Koji Tamura

**Affiliations:** 1 Department of Biological Science and Technology, Tokyo University of Science, 6-3-1 Niijuku, Katsushika-ku 125-8585, Tokyo, Japan; 2 Research Institute for Science and Technology, Tokyo University of Science, 2641 Yamazaki, Noda 278-8510, Chiba, Japan

**Keywords:** kissing-loop interactions, ligase ribozyme, hammerhead ribozyme, RNA evolution

## Abstract

The acquisition of functions via the elongation of nucleotides is an important factor in the development of the RNA world. In our previous study, we found that the introduction of complementary seven-membered kissing loops into inactive R3C ligase ribozymes revived their ligation activity. In this study, we applied the kissing complex formation-induced rearrangement of RNAs to two nonfunctional RNAs by introducing complementary seven-membered loops into each of them. By combining these two forms of RNAs, the ligase activity (derived from the R3C ligase ribozyme) as well as cleavage activity (derived from the hammerhead ribozyme) was obtained. Thus, effective RNA evolution toward the formation of a life system may require the achievement of “multiple” functions via kissing-loop interactions, as indicated in this study. Our results point toward the versatility of kissing-loop interactions in the evolution of RNA, i.e., two small nonfunctional RNAs can gain dual functions via a kissing-loop interaction.

## 1. Introduction

The “RNA world” hypothesis [1] was proposed based on the discovery made regarding catalytic RNAs (ribozymes) [2,3]. This discovery has changed the concepts of the origin of life and evolution. The RNA world provided an answer to the so-called “the chicken or the egg” problem (“DNA makes proteins that are made by DNA”). In addition to this, an experiment carried out by Noller and coworkers [4], and the structure of a ribosome peptidyl transferase center composed of RNAs entirely [5,6], enhanced the reliability of the RNA world hypothesis in biological evolution.

**Figure 1:**
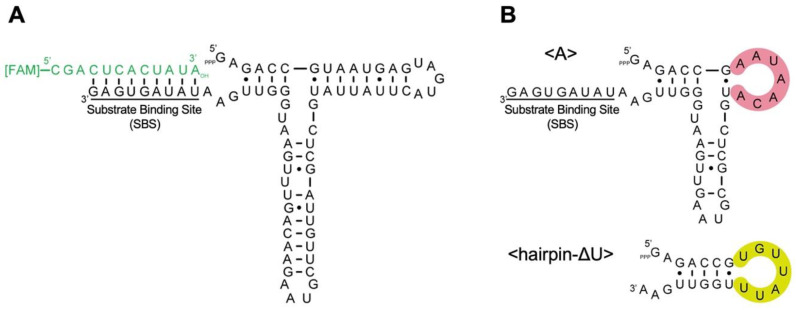
(**A**) Composition of the R3C ligase ribozyme (73-mer, black) and 5′-6-FAM-labeled ligation substrate (12-mer, green). The substrate binding site (SBS) is underlined. (**B**) Composition of <A> (49-mer, top) and <hairpin-ΔU> (22-mer, bottom). Complementary 7-membered single-stranded nucleotides (5′-AAUAACA-3′ and 5′-UGUUAUU-3′) are colored in pink and bright green, respectively. Only <A> possesses the substrate binding site (SBS).

On the other hand, the acquisition of functions via the elongation of nucleotides is an important factor in the development of the RNA world. Functional association between two RNA molecules has been shown to generate active ribozymes [7,8,9]. Furthermore, in the process of elucidating the “back to life” mechanism of inactive shortened R3C ribozymes (Figure 1) [10,11], we discovered that the introduction of complementary seven-membered kissing loops revives their ligation activity [10,11]. This phenomenon suggests that a kissing complex formation induces a functional *trans*-rearrangement of two nonfunctional shortened R3C ribozymes [12,13]. The acquisition of a function through a kissing-loop interaction-induced rearrangement is not limited to the case of R3C ribozymes. We also demonstrated that a kissing-loop interaction between *trans*-molecules produced a unique G:U wobble base pair in an appropriate position, corresponding to the acceptor stem of tRNA [14], which can be a critical recognition site for alanyl-tRNA synthetase (AlaRS) [15,16]. Therefore, two small nonfunctional RNAs can gain a function through a kissing-loop interaction.

However, the effective evolution of RNA towards the formation of a life system may require the acquisition of “multiple” functions via a kissing-loop interaction. In the present study, we demonstrate that two nonfunctional RNAs can gain “two” separate functions through a kissing-loop interaction-induced rearrangement; we also discuss the versatility of kissing-loop interactions in the evolution of RNA.

## 2. Materials and Methods

### 2.1. Preparation of RNAs

**Figure 2:**
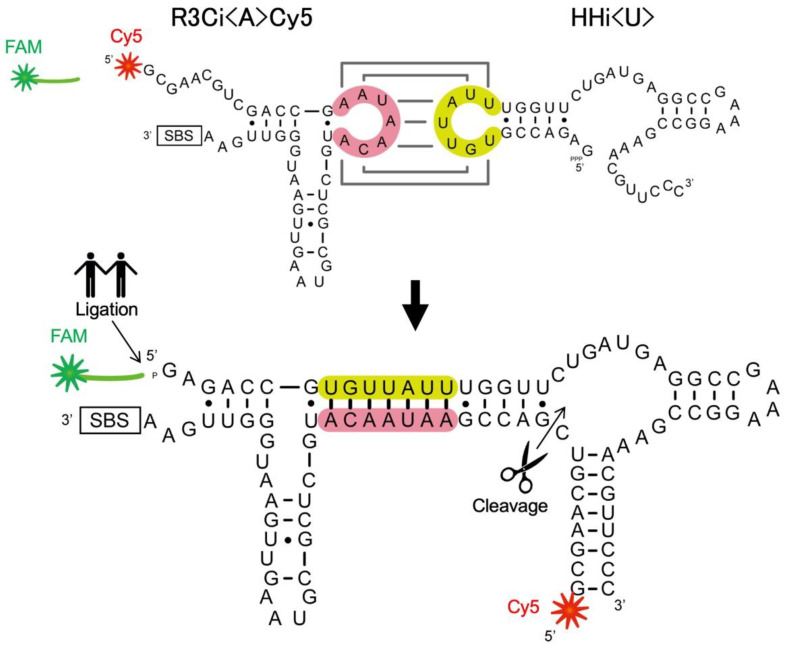
Top: Composition of R3Ci<A>Cy5 (56-mer), HHi<U> (49-mer), and the 5′-6-FAM-labeled ligation substrate in an abbreviated form (indicated with a green star and line). The 5′-terminus of R3Ci<A>Cy5 is labeled as Cy5 (indicated by a red star) and the substrate binding site (SBS) is also drawn in an abbreviated form (the detailed sequence is shown in Figure 1). Bottom: Conformational rearrangement induced by the kissing-loop interaction possibly produces not only a substrate-ligated molecule of HHi<U> (indicated by a holding-hands mark), but also a cleaved molecule by the putative hammerhead ribozyme, which would release 5′-Cy5-labeled fragments (9-mer, indicated by a scissors mark).

Non-fluorescence-labeled RNAs were prepared by transcription using T7 RNA polymerase. PCR-amplified DNAs carrying the T7 promoter and the targeted sequences were used for RNA transcription. The primers and templates were synthesized by Eurofins Genomics K.K. (Tokyo, Japan). RNA transcription was performed at 37 °C for 16 h in a reaction mixture containing 40 mM Tris-HCl (pH 8.0), 10 mM dithiothreitol, 2 mM spermidine, 8 mM MgCl_2_, and 2.5 mM each of NTP, template DNA (~0.2 mg/mL), and pure T7 RNA polymerase (~100 μg/mL) [17,18]. The transcripts were purified by denaturing 12% polyacrylamide gel electrophoresis. The concentrations of the obtained purified RNA were determined from the ultraviolet absorbance at a wavelength of 260 nm using an Implen NanoPhotometer (München, Germany). High-performance liquid chromatography-purified 5′-terminal 6-FAM-labeled RNA (12-mer) (Figure 1A) and Cy5-labeled RNA (R3Ci<A>Cy5: 56-mer) (Figure 2) were prepared by Japan Bio Services Co., Ltd. (Saitama, Japan).

### 2.2. Electrophoretic Mobility Shift Assay (EMSA)

**Figure 3:**
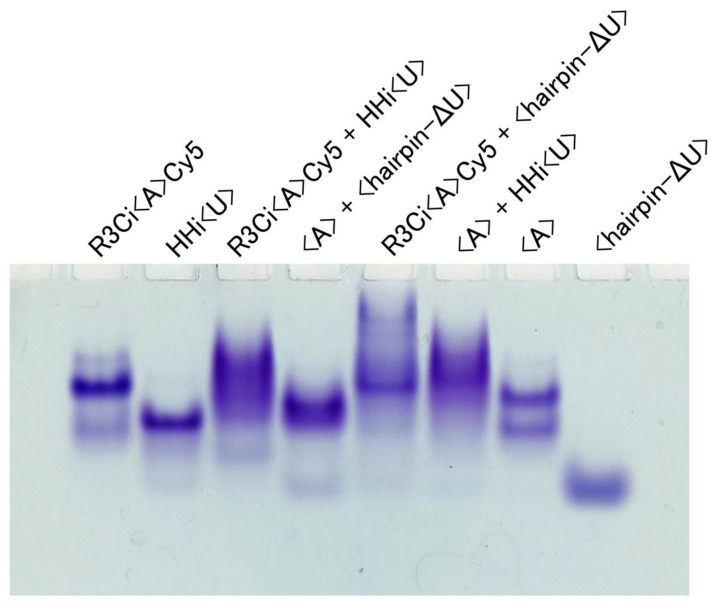
Electrophoretic mobility shift assay of various RNAs. From left to right: R3Ci<A>Cy5, HHi<U>, R3Ci<A>Cy5 with HHi<U>, <A> with <hairpin—ΔU>, R3Ci<A>Cy5 with <hairpin-ΔU>, <A> with HHi<U>, <A>, and <hairpin-ΔU>. The RNAs were separated via 8% native PAGE. The gel was stained with 0.04% toluidine blue.

An amount of 15 µL of 30 µM of the combination of RNA(s) shown in Figure 3 in R3C buffer (50 mM Tris-HCl (pH 8.5) and 15 mM MgCl_2_) was heated at 37 °C for 5 min and then incubated at 23 °C for 18.5 h. After the addition of loading buffer containing glycerol, the solution was analyzed by electrophoresis through nondenaturing 8% polyacrylamide gels in R3C buffer. The gel was stained with 0.04% toluidine blue [12,14,18].

### 2.3. Analysis of Ligation and Cleavage Activity

**Figure 4:**
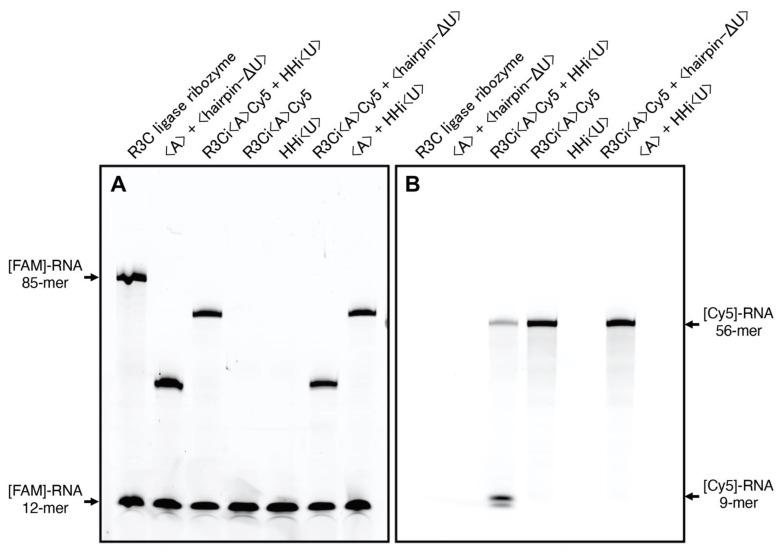
(**A**) Ligation activities using R3Ci<A>Cy5, HHi<U>, <A>, and <hairpin-ΔU>. Green bands (λ_ex_ = 473 nm, Y520 filter) associated with 6-FAM labeled RNAs were detected. (**B**) Cleavage activities using R3Ci<A>Cy5, HHi<U>, and <hairpin-ΔU>. Red bands (λ_ex_ = 635 nm, R670 filter) associated with Cy5-labeled RNAs were detected.

**Figure 5:**
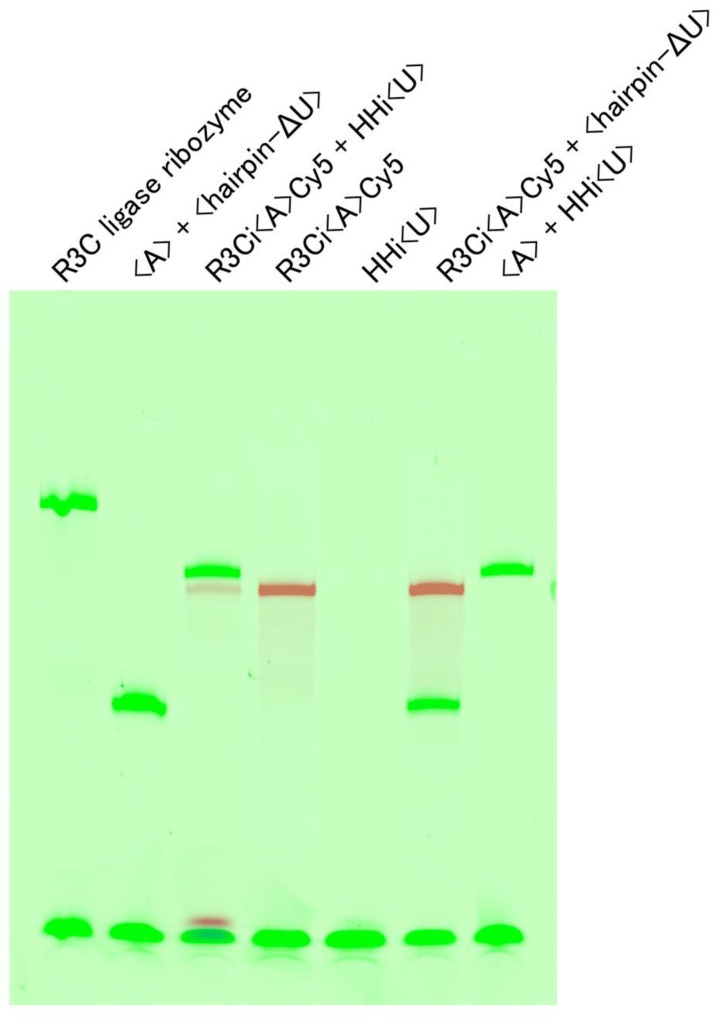
Visualization of ligation and cleavage activities using R3Ci<A>Cy5, HHi<U>, <A>, and <hairpin-ΔU>. The image was obtained by merging the photos obtained in Figure 4.

**Figure 6:**
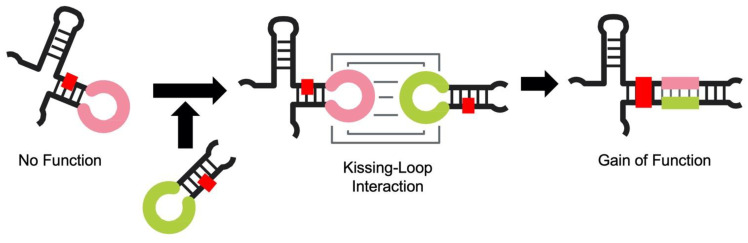
A simplified schematic representation of the evolutionary processes involved in functional RNA formation. Conformational changes mediated by multiple kissing-loop interactions, which started from short hairpin RNAs, possibly contributed to the effective evolution of RNA toward the formation of a life system.

The reaction was performed according to the method of Rogers and Joyce with a slight modification [10,11,12,13]. The combination of 5 µM RNA(s) shown in Figure 4 dissolved in a solution containing 50 mM Tris-HCl (pH 8.5) and 15 mM MgCl_2_ was first heated to 37 °C for 5 min and then cooled to 4 °C. Then, the reaction was started by adding 1.5 µL of 50 µM 6-FAM-labeled substrate to 13.5 µL of the solution. The final concentrations of each RNA shown in Figure 4 and 6-FAM labeled substrate were 5 µM each. After incubation for 18.5 h at 23 °C, 15 ΔL of 7 M urea and 0.08% (*w/v*) bromophenol blue were added. The solution was then applied to a denaturing 12% polyacrylamide gel for electrophoresis. The gel was analyzed on a Typhoon FLA 7000 system (GE Healthcare Japan, Tokyo, Japan) using a Y520 filter (for 6-FAM) and R670 filter (for Cy5). The merging of the photo images was performed using graphics software (Adobe Photoshop) (Figure 5).

## 3. Results

Our previous study showed that the truncation of the R3C ligase ribozyme resulted in a marked decrease in ligation activity (Figure 1) [11]. One such truncated mutant with a seven-membered single-stranded nucleotide (5′-AAUAACA-3′) is <A> (49-mer) (Figure 1B) [12,13]. <hairpin-ΔU> (22-mer) is a mutant of the R3C ligase ribozyme with a complementary seven-membered single-stranded nucleotide (5′-UGUUAUU-3′), but with no “grip” and no substrate-binding site (SBS) (Figure 1B) [12,13]. R3Ci<A>Cy5 (56-mer) is a 5′-Cy5-labeled mutant with extra nucleotides at the 5′-terminus of <A> (Figure 2). HHi<U> (49-mer) is designed based on the hammerhead ribozyme [19,20] with the same seven-membered single-stranded nucleotide (5′-UGUUAUU-3′) as <U> (Figure 2).

### 3.1. Electrophoretic Mobility Shift Assay (EMSA)

The native gel electrophoretic characterization showed that <hairpin-ΔU> alone behaved as a monomer, but <A> alone behaved as both a monomer and dimer (Figure 3). However, the combination of <A> and <hairpin-ΔU> showed the major formation of a hetero-dimer (Figure 3), which could be derived from the kissing-loop interaction-induced rearrangement, consistent with our previous results [13]. Using these positions as controls, we found that the combinations of R3Ci<A>Cy5 and HHi<U>, R3Ci<A>Cy5 and <hairpin-ΔU>, and <A> and HHi<U> also formed dimers (Figure 3).

### 3.2. Ligation Activity towards 6-FAM Labeled Substrates

We detected green bands (λ_ex_ = 473 nm, Y520 filter) associated with 6-FAM-labeled RNAs on a denaturing 12% polyacrylamide gel electrophoresis, and used the ligation activity of the R3C ligase ribozyme as a positive control (Figure 4A). The combination of <A> and <hairpin-ΔU> exhibited apparent ligation activity for the 6-FAM-labeled substrate; it was ligated to <hairpin-ΔU> as previously reported (Figure 4A) [13]. Although R3Ci<A>Cy5 alone and HHi<U> alone did not show any ligation activity towards the 6-FAM labeled substrate, a combination of R3Ci<A>Cy5 and HHi<U> produced the ligated band, the length of which was much longer than that of the combination of <A> and <hairpin-ΔU> (Figure 4A). The combination of R3Ci<A>Cy5 and <hairpin-ΔU> also produced a ligated product, and the band length was almost the same as that produced by the combination of <A> and <hairpin-ΔU>, suggesting that the 6-FAM-labeled substrate was ligated to <hairpin-ΔU>. A combination of <A> and HHi<U> also ligated the 6-FAM-labeled substrate, and the position of the band was almost the same as that produced by the combination of R3Ci<A>Cy5 and HHi<U> (Figure 4A), suggesting that the 6-FAM-labeled substrate was ligated to HHi<U>.

### 3.3. Cleavage of R3Ci<A>Cy5 by the Expected Hammerhead Ribozyme

On the same denaturing 12% polyacrylamide gel shown in Section 3.2, we detected red bands (λ_ex_ = 635 nm, R670 filter) associated with Cy5-labeled RNAs (Figure 4B). The original Cy5-labeled RNA is only R3Ci<A>Cy5, and the combination of R3Ci<A>Cy5 and HHi<U> caused the decrease in R3Ci<A>Cy5 (56-mer) and the production of the 5′-Cy5-labeled fragment (9-mer) under the experimental conditions (Figure 4B). However, the combination of R3Ci<A>Cy5 and <hairpin-ΔU> did not show any cleavage of R3Ci<A>Cy5 (Figure 4B). The super-positioning of the green and red band images on the same gel clearly showed the acquisition of the two expected functions via the kissing-loop interaction (Figure 5).

## 4. Discussion

Both R3Ci<A>Cy5 and HHi<U> are nonfunctional RNAs; this changed by introducing complementary seven-membered loops to each of them (Figure 2). By combining R3Ci<A>Cy5 and HHi<U>, both ligase activity (derived from the R3C ligase ribozyme) and cleavage activity (derived from the hammerhead ribozyme) were obtained. The experimental design made it possible to detect the cleavage activity via the putative hammerhead ribozyme derived from the conformational rearrangement of R3Ci<A>Cy5 and HHi<U> through a kissing-loop interaction. The 5′-terminus of R3Ci<A>Cy5 was labeled as Cy5; the hammerhead ribozyme could cleave between C9 and G10 and produce a 5′-Cy5 labeled fragment (Figure 2).

The RNA world is a plausible stage in the Earth’s history when lifeforms used RNA to form enzymes and genomes [1]. The RNA world is not a form of life; however, the system based on it must surely have existed in the early stages of biological evolution. The reason for such speculation is that RNA can be a potential form of self-replication, although the perfect self-replication of RNAs has not yet been attained [21]. It has been reported that peptides can replicate under certain conditions [22]; however, the interactions represented by the Watson–Crick base pairs are general notable features seen only in nucleotides. In such cases, the formation of longer RNAs is inevitable in terms of the acquisition and improvement of functional RNAs. In addition to this, the peptidyl transferase center (PTC) in ribosomes is composed of two symmetrically arranged tRNA-like units [21,23,24,25,26]. Recently, it was found that certain combinations of the aforementioned segments are capable of mediating peptide bond formation [27] using chemically synthesized P- and A-site analogs [28]. Furthermore, we have shown that a piece of RNA formed a dimer and that a peptide bond was formed between two aminoacyl minihelices (primordial tRNAs) tethered by the dimeric structure [29].

Hairpin RNAs are the most fundamental form of short RNAs [21,30,31] and the minihelix (hairpin structure composed of a coaxial stack of the acceptor stem on the T-stem of tRNA) is thought to be a progenitor of modern tRNA [21,32,33]. In addition to this, a hairpin RNA with NCCA-3′ may be related to the origin of homochiral aminoacylation in the RNA world [21,34,35,36,37]. Hairpin RNAs are composed of a stem and loop; the loop region is the most plausible place for interactions between different molecules. In fact, a kissing-loop interaction between two nonfunctional independent molecules can induce a conformational change and produce one function (e.g., the formation of a G:U wobble base pair corresponding to G3:U70 in tRNA^Ala^ [14], which is the major identity determinant by AlaRS [15,16]). The present study clearly emphasizes that a kissing-loop interaction between two nonfunctional independent molecules produces dual functions simultaneously.

As shown in previous reports regarding kissing-loop interactions [13,38,39,40], and also assumed in the present study, loops with six to seven nucleotides can facilitate the kissing-loop-mediated conformational changes. Although heterogeneous combinations of six- and seven-membered loops, or combinations of loops with fewer than six members, generally have difficulty with the kissing-loop-mediated conformational changes due to thermodynamic constraints [13], sequence diversity is allowed, and many RNA loops are likely to have ample opportunities to take advantage of such interactions in the process of evolution. For structural remodeling by kissing-loop interactions, further consideration would be possible [41,42].

Regarding “two ribozyme functions from one RNA” and “evolution of RNA by adding nucleotides”, DasGupta et al. also showed that a single sequence could harbor two distinct active sites and perform both hairpin ribozyme and hammerhead ribozyme activities. They also proposed RNA evolution by nucleotide accretion [43]. *Trans*-molecular structural changes due to kissing-loop interactions utilized in our present study are a familiar phenomenon in biological systems, e.g., in HIV dimerization [44,45,46,47,48]. Therefore, our results show the importance of the concept that kissing-loop interactions can lead to the acquisition of “multiple” functions in the RNA world (Figure 6).

In terms of accurate replication under Darwinian selection in enzyme-free nucleotide polymerization, Eigen and Schuster insisted that the nucleotide length is no more than approximately 100 when the physical properties inherent to current nucleic acids are considered [49]. In addition, RNA synthesis in a clay mineral (montmorillonite) environment has been shown to be at most ~50-mer [50]. On the other hand, the minimized L1 ligase ribozyme only contained a catalytic core of ∼35-mer [51,52]. A complete library consisting of one copy each of all 1.18 × 10^21^ possible 35-mers would weigh only approximately 22 g (the mass of the Earth is approximately 6 × 10^24^ kg). Therefore, when considering the effects of ligation and time scale, it does not necessarily seem to be impossible that the two RNAs used in our study could be synthesized via random processes on primitive Earth.

**Figure 7:**
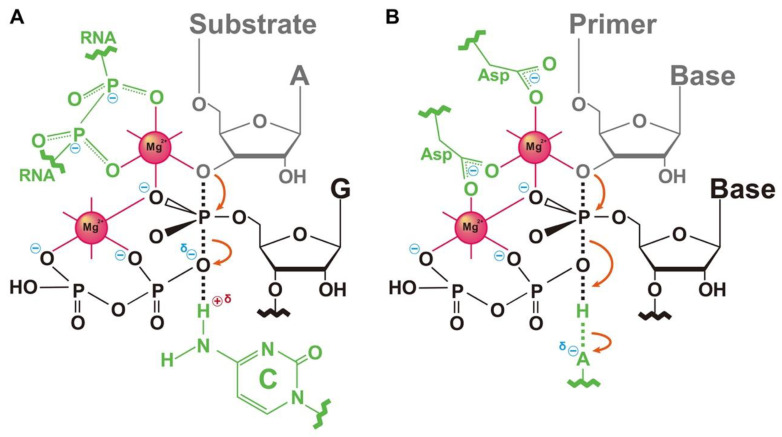
Transition-state-stabilization model constructed from either RNA or protein. Active site of (**A**) class I ligase ribozyme and (**B**) RNA polymerase. This figure was modified from Shechner et al. [55].

Finally, we comment on the transition from the RNA world to the protein theater. Among some in vitro evolved ligase ribozymes, the class I ligase ribozyme has a catalytic rate of the order of 100 cycles per minute [53,54], which is comparable to that found in natural proteinaceous polymerases. In fact, a transition-state-stabilization model constructed on the basis of the crystal structure of the ribozyme [55] and proteinaceous polymerases [56,57] is similar (Figure 7). Thus, the transition from ribozymes to protein enzymes may have occurred while maintaining evolutionary continuity in terms of tertiary structure.
